# Applications of Azide-Based Bioorthogonal Click Chemistry in Glycobiology

**DOI:** 10.3390/molecules18067145

**Published:** 2013-06-19

**Authors:** Xiu Zhang, Yan Zhang

**Affiliations:** Ministry of Education Key Laboratory of Systems Biomedicine, Shanghai Center for Systems Biomedicine (SCSB), Shanghai Jiao Tong University, 800 Dong Chuan Road, Minhang, Shanghai 200240, China; E-Mail: zhangxiu@sjtu.edu.cn

**Keywords:** click chemistry, azide, bioorthogonal, glycosylation, glycobiology

## Abstract

Click chemistry is a powerful chemical reaction with excellent bioorthogonality features: biocompatible, rapid and highly specific in biological environments. For glycobiology, bioorthogonal click chemistry has created a new method for glycan non-invasive imaging in living systems, selective metabolic engineering, and offered an elite chemical handle for biological manipulation and glycomics studies. Especially the [3 + 2] dipolar cycloadditions of azides with strained alkynes and the Staudinger ligation of azides and triarylphosphines have been widely used among the extant click reactions. This review focuses on the azide-based bioorthogonal click chemistry, describing the characteristics and development of these reactions, introducing some recent applications in glycobiology research, especially in glycan metabolic engineering, including glycan non-invasive imaging, glycomics studies and viral surface manipulation for drug discovery as well as other applications like activity-based protein profiling and carbohydrate microarrays.

## 1. Introduction

Glycosylation, the most complex and ubiquitous post-translational modification, is involved in a number of physiological and pathological processes. In living systems, more than half of the proteins are glycosylated [1]. Glycans serve as recognition molecules, affect cell proliferation and differentiation, and regulate immune response, inflammation, and cancer metastasis. Glycans on the cell surface displayed a unique signature of certain cell type and different life status. Changes in glycan profile are often associated with diseases. In 1988, Oxford University Professor De Weike wrote an overview of “Glycobiology” in *Biochemistry Annual Reviews*, which marks the birth of the new branch of glycobiology [2]. However, for many years researchers have suffered from the lack of advanced tools to explore the functions of glycan. Since the biosynthesis of glycan is not template driven, its structure is heterogeneous and hard to engineer by conventional genetic ways, and consequently, the development of glycosylation research is still lagging behind that of genes and proteins.

As one of the new emerging approaches, chemical glycobiology is accelerating the pace of glycobiology study by bringing efforts to elucidate and manipulate the functional roles that glycans play in biological processes. The chemical technologies are able to overcome the chemical similarity and structural complexity of glycoconjugates. Among all those chemical tools, the bio-orthogonal reagents of click chemistry have demonstrated unique advantages: bioorthogonality, biocompatibility, high efficiency and high specificity. The term “bioorthogonal click chemistry” was first coined by Carolyn Bertozzi in 2003 [3]. It refers to any chemical reaction that can occur inside living systems without interfering with native biochemical processes. The term “bioorthogonal” here refers to the quality of being absent from all naturally biosystems, while remaining reactive in water solutions. Prescher and Bertozzi defined bioorthogonal click chemical reporters as “non-native, non-perturbing chemical handles that can be modified in living systems through highly selective reactions with exogenously delivered probes ” [4].

**Figure 1:**
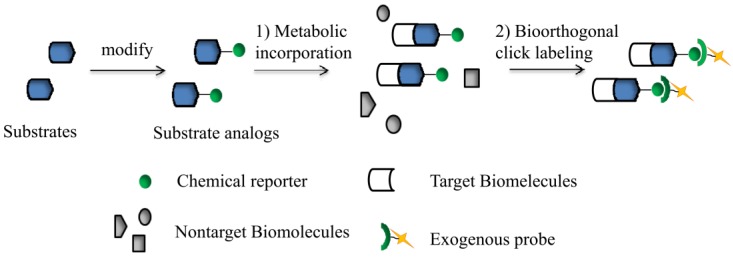
Two-step bioorthogonal chemistry strategy. (1) A bioorthogonal functional moiety (chemical reporter) was installed into substrates (blue shapes) to generate substrate analogs which can selectively incorporated into the target biomolecules (white shapes) through metabolism; (2) Chemical reporters selectively covalent with exogenous probes by click reaction for further detection and isolation.

Bioorthogonal click chemistry is a two-step reaction and needs a pair of functional groups (Figure 1). First, the bioorthogonal functional moiety (chemical reporter) of a compound is incorporated into a substrate. Second, the reporter is covalently linked to an exogenous probe through a click reaction, which allows for detection and isolation of the target. Most importantly, the covalent reaction between these two compounds needs to proceed rapidly and selectively in a physiological environment with biocompatible pH (6–8), and temperature (37 °C), have less byproducts and be nontoxic. The chemical reporter should be inert *in vivo*, have no reaction with the biological environment and small enough to modify the target substrate without any functional and spatial interference. Therefore, so far, only a handful of bioorthogonal click reagents have been developed for biological use [4]. 

During the development of glycobiology, the most influential and most widely used bioorthogonal click reagent is azide, a rather small molecule with attractive bio-stability and non-perturbation ability. It can modify not only proteins, but also all classes of biomolecules, including nucleotides, lipids, and glycans, as well as other metabolites. Once installed into a target substrate, azides can covalently combine to its complementary partner by azide-alkyne cycloaddition (Cu-catalyzed or strain promoted) or Staudinger ligation. Especially in glycobiology research, the azide and alkyne couple was widely used in metabolic engineering and monosaccharide labeling. To date, azide more than just plays a role as the bioorthogonal chemical reporter, it also can be an elite glycan handle applied to various glycobiology research topics.

### 1.1. Azide-Based Click Chemistry in Glycobiology

There are mainly two modes of reactivity of azide-based biorthogonal click chemistry that have been developed in glycobiology research [5,6,7]. One is a 1,3-dipole [3 + 2] cycloaddition with alkynes, which can be further divided into copper-catalyzed azide-alkyne cycloaddition (CuAAC) and strain-promoted alkyne-azide cycloaddition (SPAAC). The other type is azide-Staudinger ligation. 

#### 1.1.1. Azide-Alkyne Cycloaddition

The most primitive azide-alkyne cycloaddition was thoroughly explored by Huisgen and coworkers in the 1950–70s. Although the reaction is highly exothermic and experienced very low reaction rates and yields, it had a great impact on further research. The high pressures and temperatures required in this reaction were the biggest constraint for its application in living systems. It didn’t undergo enormous changes until the use of copper as the catalyst solved this issue.

The term copper-catalyzed azide-alkyne cycloaddition (CuAAC), a widely well known example of click chemistry, was coined by Sharpless in 2002 [8], it was described as a better version of Huisgen’s [2 + 3] cycloaddition with little solvent dependence and better adherence of click chemistry principles [9]. The presence of copper greatly changed the reaction mechanism and improved the yields, dramatically accelerated the cycloaddition reaction, and most significantly, it can be performed below room temperature. It has been widely used to make covalent connections between substrates tagged with functional groups such as azides or alkynes, but there is another severe restriction shouldn’t be ignored: the free Cu (I) ions are toxic to organisms. Sodium ascorbate was often used to reduce the Cu (I) oxidation state, but another reagent is also needed since the Cu/ascorbate system generates amounts of reactive species, including reactive oxygen species (ROS). Some Cu (I)-stabilizing ligands were developed to further accelerated the reaction, such as: *C3*-symmetric derivative (TBTA) [10], tris (3-hydroxypropyl-triazolylmethyl) amine (THPTA) [11]. Finn and co-workers applied THPTA combined with ascorbate in CuAAC which gave rise to a robust azide modified *N*-acetylmannosamine (Ac_4_ManNaz) label for confocal microscopy [11]. A report by Wu made a comparative study of BARAC-mediated cycloaddition and bio-benign CuAAC, newly discovered BTTAA and BTTES [12] showed a higher activity in accelerating the CuAAC. BTTAA-Cu^I^ catalyst is better for cell surface and *in vivo* azide-alkyne ligation [13].

**Figure 2:**
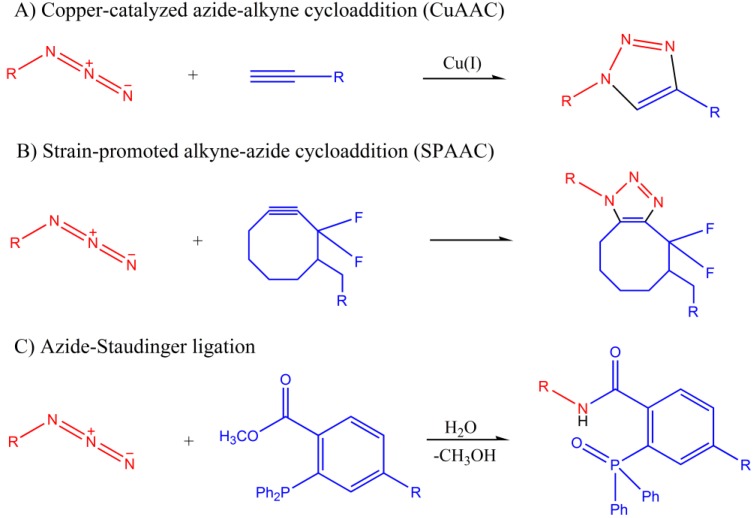
Illustration of three kinds of bioorthogonal click chemistry in glycobiology. (**A**) Copper-catalyzed azide-alkyne cycloaddition (CuAAC); (**B**) Strain-promoted alkyne-azide cycloaddition (SPAAC); (**C**) azide-Staudinger ligation.

Strain-promoted alkyne-azide cycloaddition (SPAAC) is an extension of CuAAC development (Figure 2B). Due to the toxic features of copper, which can catalyze atmospheric oxygen reactions to form ROS, labeling living cells by copper-catalyzed azide-alkyne click chemistry should be optimized. Researchers have employed strain-promoted [3 + 2] cycloaddition of azides and cyclooctynes as an alternative means for azide-alkyne activation. The Bertozzi group synthesized an improved biotinylated cyclooctyne which can directly react with azide-labeled cells with no negative effects on cell viability [14]. Nonetheless, compared to CuAAC, the copper-free click chemistry is still limited by its low rates. On the basis of the previous studies, researchers are exploring new ways to increase the rate of strain-promoted cycloadditions [15]. Different types of cyclooctyne probes, like DIFO-FLAG, DIMAC-FLAG, ALO-FLAG can be used directly to label azido sugars in living organisms [16], while for some *in vitro* studies, especially some proteomic applications, CuAAC seems to be the most efficient and convenient strategy.

#### 1.1.2. Azide-Staudinger Ligation

Azide-Staudinger ligation (Figure 2C) was first introduced by Saxon and Bertozzi in 2000 and is also known as non-traceless azide-phosphine ligation [17]. This reaction needs no catalyst and achieves high yields in water. It forms an amide bond between one nitrogen atom of the azide and triarylphosphine, which is also bioorthogonal. It proceeds readily at pH 7 and is biocompatible which made its *in vivo* applications possible. Most importantly, the Staudinger ligation meets most of the criteria required in a biologically compatible environment: rapid reactions inside the cell, no toxic byproducts and stability in water. Biotin-phosphines, FLAG-phosphines and other tag-labeled substances were designed for glycan detection using fluorescent secondary reagents (FITC-avidin and FITC-anti-FLAG antibody) [18]. Lemieux *et al*. reported a fluorogenic coumarin-phosphine dye, which can be activated by azide-Staudinger ligation [19]. It’s a significant tool for detection and quantitation of azide-labeled modifications, however, the phosphines are reducing agents, easily oxidized by air or metabolic enzymes to generate phosphine oxide byproducts. When comparing to strain-promoted cycloaddition, it exhibits better sensitivity. For studies involving live organisms, the Staudinger ligation and copper-free azide-alkyne cycloaddition seem to be the better choices.

The appealing bioorthogonal features of azide have enabled its applications in many areas of glycobiology. For example, non-invasive imaging of glycan distribution and trafficking under conditions of cell stimulation or pharmacological intervention performed in cells, tissues, and whole organisms or direct manipulation or purification of target glycoconjugates using azide handles. Here we describe some important but not all applications of azide-based bioorthogonal click chemistry in glycobiology research.

## 2. Applications of Azide-Based Click Chemistry in Glycan Research

### 2.1. Glycan Metabolic Engineering

Many biomolecules—nucleic acids, glycans, lipids—as well as various posttranslational modifications and metabolites cannot be traced by genetically encoded tags. Lectins and antibodies are first widely used to image glycan-binding proteins and for glycoconjugate detection and enrichment. Lectins are non-immune origin proteins that bind to a certain glycan structural motif and this affinity has been utilized in glycan identification, enrichment, and glycomics profiling, but their binding dissociation constants between the target oligosaccharide are so weak (10^−4^ to 10^−3^ M), that they show a lack of specificity. Unlike actins, antibodies generated against glycan structures can strongly attach to antigen binding sites of substrates, but their large size limits its application inside cells.

**Figure 3:**
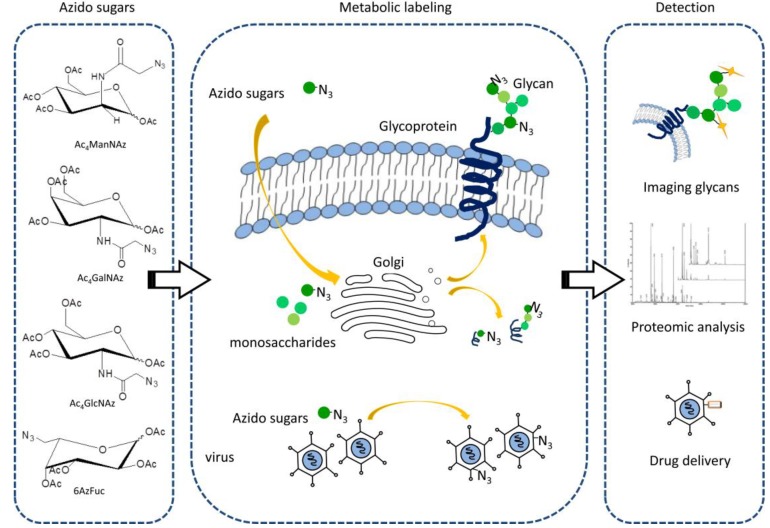
Metabolic engineering strategy. Different types of unnatural monosaccharides residues (azido sugars): *N*-azidoacetylmannosamine (ManNAz), *N*-azidoacetylglucosamine (GlcNAz), *N*-azidoacetylgalactosamine (GalNAz), and 6-azidofucose (6AzFuc) can incorporate into glycoconjugates through cells’ or virus’ own biosynthetic machinery. After displayed on cell surfaces or virus capsid, it can be applied to image glycans *in vivo*, identify targets by mass spectrometry and deliver cancer specific drugs.

Metabolic engineering offers means to manipulate glycans in cells or on cell surfaces in a defined way. It introduces unnatural monosaccharides residues into cellular glycans through cell’s own biosynthetic machinery (Figure 3). When specific types of target monosaccharides are modified by azides, the azido analoges can be recognized and accepted by cells, then transformed into substrates and transported into the Golgi compartment to format complex glycogonjugates. Through a covalent reaction with a second bioorthogonal reagent, azido sugars can be used for cellular assays.

The first chemical handle explored to monitor cell surface glycans was the ketone handle [20], but the *in vivo* existence of a large number of compounds containing ketone and aldehyde groups, which compete with endogenous keto-metabolites, has limited its application inside cells and organisms. Meanwhile, the biologically inert azide group, is an appealing moiety that can circumvent this problem. 

Unlike antibodies or lectins, chemoselective ligation *in vivo* acts more like a bridge between cells and artificial synthetic substances engineered to manipulate biological processes. This has enabled non-invasive imaging of glycans, and introduced a handle for glycomic analysis as well as cancer targeting drug delivery.

There are mainly fours subtypes of azido sugars have been synthesized and developed for glycan metabolic engineering: *N*-azidoacetylmannosamine (ManNAz), *N*-azidoacetylglucosamine (GlcNAz), *N*-azidoacetylgalactosamine (GalNaAz), and 6-azidofucose (6AzFuc).

#### 2.1.1. Metabolic Labeling for *in Vivo* Imaging of Glycans

For glycobiology research, the need for imaging glycans in their native conditions seems increasingly urgent. Metabolic labeling with bioorthogonal chemical reporters is an alternative way for glycan imaging. GFPs are relatively large and can cause significant structural perturbation, while chemical reporters like azide are small enough to cause less interference to glycostructures through a metabolic pathway. Azide-reactive bioorthogonal probes exhibit exquisite chemoselectivity and can be used for probing the distribution, abundance and dynamic changes of glycans *in vivo*. Like the principles described earlier, the process of imaging glycans entails two steps (Figure 3). First, living cells or organisms are incubated with azide-adorned analog precursors, which can be utilized by cells’ metabolic pathways without interfering. Once azido glycans are incorporated into a target, it can selectively bind covalently with a second probe containing a complementary bioorthogonal moiety with an imaging probe. 

For cell surface glycan imaging, azido sugars can directly added to the culture medium to treat cells, and then detected by fluorescent probes or analyzed by flow cytometry. After various cell types have been imaged, the model can be extended to living animals which can provide a more informative view of the biological effects of glycans. Injecting an appropriate amount of azido analogs into mice provides a robust labeling of target glycoproteins. Azido sugars have been utilized to visualize glycans in different cell types [21] and living organisms, such as mice [22,23] and zebrafishes [24,25,26]. More detailed reviews about chemical glycan probing has been provided before [7], The first time azide was used as a chemical handle in living systems was for probing sialic acid on cell surfaces [17]. Cells were treated with preacetylated *N*-azidoacetylmannosamine (Ac_4_ManNAz) for 3 days, then reacted with biotinylated phosphine by Staudinger ligation, and analyzed by flow cytometry followed by staining with fluorescein isothiocyanate (FITC) avidin. A substituted difluorinated cyclooctyne (DIFO) can be used to indentify sialoglycoconjugates with rapid internalization kinetics in cells and mice. Different DIFO-fluorophore conjugates also enabled two-color, time-resolved labeling of glycan trafficking during early zebrafish embryogenesis [26]. 

In accordance with the same strategy, peracetylated *N*-azidoacetylglucosamine (Ac_4_GlcNAz) can be processed by the hexosamine salvage enzymes and labels *O*-GlcNAc modified glycoproteins in the nucleus and cytoplasm [27]. Inner protein *O*-GlcNAcylation is competing with phosphorylation for Ser/Thr sites which could dramatically change the functions of target proteins. The most dominant form of *O*-glycosylation is mucin-type *O*-linked glycosylation, where *N*-acetylgalactosamine (GalNAc) is attached to a Ser/Thr residue of the polypeptide in the *α*-anomeric configuration. Some reports have revealed that when treated living systems with Ac_4_GalNAz, it more like to label *O*-GlcNacylated proteins than GalNAcylated protein itself [28]. This most likely due to the rate-limiting step in GlcNAc salvage pathway: pyrophosphorylase. GalNAz transforms to GlcNAc in a C_4_ epimerase-dependent manner, which made the overall GalNAz labeling efficiency very low. On the other side, GalNAz can be efficiently converted to GlcNAz by GalE (UDP-GlcNAc/GalNAc C_4_-epimerase). This underlies that Ac_4_GalNAz could be used to label *O*-GlcNAcylated proteins as well as further analysis, but in complicated studies of Ac_4_GalNAz-labeled glycoproteins in particular. A feasible approach is to purify nuclear and cytoplasmic glycoproteins separately by simple subcellular fractionation.

Fucosylated glycans can metabolically labeled by using peracetylated 6-azidofucose (6AzFuc) or peracetylated 6-alkynylfucose (alkynyl fucose) through the fucose salvage pathway. FucAz successfully traverses the fucose salvage pathway and is expressed into cell surface glycans in cultured mammalian cells, but shows low metabolic efficiency [29,30]. A newly reported azide-functionalized analogue of GDP-fucose (GDP-FucAz) bypassed the salvage pathway and is accepted by fucosyl-transferases, and then exhibited robust cell surface labeling of fucosylated glycans during zebrafish development [31,32].

The metabolic labeling by click chemistry has provided a new method for the imaging of glycans in living systems. However, since the azido sugars are analogs, their affinity to target enzymes is lower compared to endogenous sugars. *In vitro* studies have revealed the *V*_MAX_/*K*_M_ value for UDP-GalNAz as a substrate for ppGalNAc-T was 0.2 relative to that for UDP-GalNAc [33]. For *in vivo* metabolic labeling, labeling efficiency varies among different cell types, and is determined by some key enzymes, such as UDP-Glc/GlcNAc C_4_-epimerase [34]. The recognition of the enzymes on the azido sugars ultimately determines the labeling efficiency and then decides whether it can fully reflect the *in vivo* glycan biological distribution. For glycan imaging, this strategy is still the optimal method with less perturbation.

#### 2.1.2. Glycan Enrichment and Glycomics Analysis

The most important issue for complex glycan structural and functional analysis is to develop high-throughput and robust technologies. Epitope tags, like biotin, FLAG, myc and His_6_ facilitate detection and enrichment of azide-tagged proteins.

Ac_4_GlcNAz allows detection of the *O-*GlcNAc metabolism mechanism and its participation in diverse intracellular functions. When using a biotinylated phosphine capture reagent, azido-GlcNAc modified proteins were isolated and detected by nano-HPLC-MS/MS [35]. Using a chemoenzymatic method coupled with IGOT-LC-MS analysis, Narimatsu and co-workers identified and site mapped fucosylated *N*-glycans [31]. Moreover, after *in vivo* metabolic labeling, trifunctional probes with an alkyne-tagged group, click-activited fluorescent and biotin moieties enabling glycan enrichment and have been developed for glycoproteomic researches [36]. 

An alternative method is the chemoenzymatic strategy developed by the Hsieh-Wilson group [37,38]. Treatment of cell lysates with a mutant galacytosyltranferase (β4GalT1) which can transfer the UDP-azidogalactose substrate (UDP-GalNAz) to *O*-GlcNAcylated proteins. Coupling azide-alkyne chemistry, this strategy has been used to mapping *O*-GlcNAc modified proteins, and it also can be utilized to indentify other types of *O-*glycans and *N*-glycans. 

**Figure 4:**
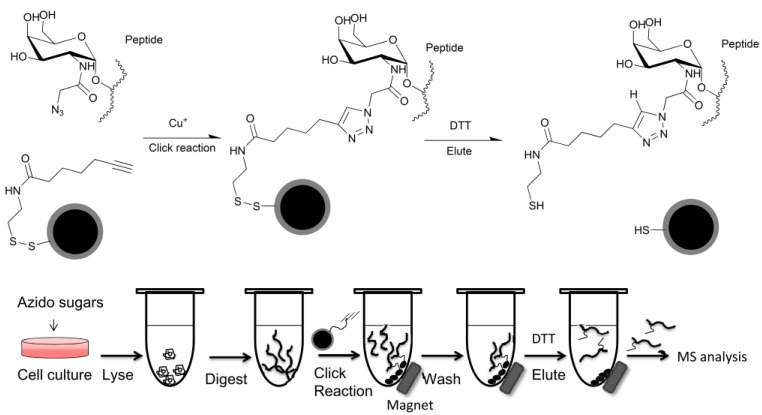
Capture strategy of disulfide and terminal alkyne modified magnetic silica particles (DA-MSPs). DA-MSPs captures azido peptides from biological samples via click chemistry between the alkyne of DA-MSPs and the azide of metabolic labeled peptides.

Recently, our group has developed a kind of disulfide and terminal alkyne-modified magnetic silica particle, which can specifically capture azide-tagged peptides from nonspecific albumin peptides from a bovine serum albumin (BSA) mixture (Figure 4) [39]. This underlies the potential use of these magnetic particles for enrichment of target glycan modified peptides from cell lysates, tissue extracts or sera. When combined with nano-HPLC-mass or mass spectrometry it can identify potential glycan-modified glycoproteins. Because most glycosylation pattern changes are correlated with cancer progression, all these azido analogues are potential tools for cancer biomarker discovery. Designing nanoparticles based on click chemistry would provide more materials and improvement for glycoconjugates enrichment.

#### 2.1.3. Viral Surface Engineering and Drug Delivery

Previous studies have introduced the modification of azides or alkynes on viruses’ surfaces or virus-like particles [40,41]. These handles could be excellent spots for imaging and drug delivery applications (Figure 3). Adenoviral particles are first metabolically labeled with GlcNAz and delivered to a virus capsid with no loss in viral production or infectivity [42]. Then they are covalently linked to a cancer-targeting motif: folate-containing alkyne probe. This two-step process provides adenoviruses chemoselectively modified with facile appendages, such as cancer targeting motifs or functional molecules. Conditionally replicative oncolytic vectors designed to preferentially replicate in cancerous cells, when combined with chemotherapeutics such as paclitaxel/decetaxel, demonstrate significant synergism cell toxicity. This incorporation occurred between an azide installed on the virus’ surfaces and alkyne-modified paclitaxel [43]. The chemoselective modification takes advantage of both oncolytic vectors’ cancer targeting capability and the toxicity of paclitaxel. Thus, azide-alkyne cycloaddition could provide a non-canonical and bio-compatible linker for virus metabolic engineering for cancer therapy.

### 2.2. Other Applications

#### 2.2.1. Click Chemistry-Based Activity Based Protein Profiling (CC-ABPP)

**Figure 5:**
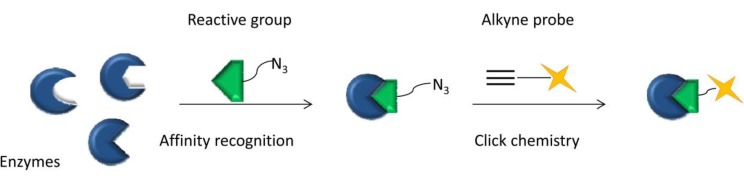
Activity based protein profiling strategy. A designed reactive group can specifically bind to target enzymes’ active site. When covalently linked to alkyne probes, it can be further detected by high-throughput technology.

Glycosylation of glycoconjugates occurs in the Golgi and endoplasmic reticulum by various glycosyltransferases and glycosidases. In response to different biological stimulations, glycan structures are remodeled, degraded, and *de novo* synthesized constantly. Studying the dynamic activities of these enzymes is particularly important. Activity-based protein profiling (ABPP) is an emerging functional proteomics technology to monitor enzyme activity [44], but the bulky reporters have limited it’s *in vivo* applications. Click chemistry-based strategies for ABPP (CC-ABPP) provide a valuable complement to standard methods of ABPP [45]. The activity-based probes contain two elements: an azide-tagged reactive group that is covalently linked to the active site in the enzyme and the other group are reporter tags for detection or purification (Figure 5). When proteomes are treated with chemical probes, it only labels active enzymes, but not those lacking complementary domains or those inhibited by regulators. Building upon the features of azide-alkyne cycloaddition, the inert coupling partners enable the reporter to attach the probe after enzymes have been labeled and thus achieve the identification of targets of enzyme activities expressed in living systems. 

Some glycosidases enzyme families were successfully modified by ABPP [46] and Staudinger ligation-based ABPP have also been reported for *in vivo* profiling of the proteasome [47]. The weak substrate affinity and low catalytic proficiency of some glycosyltransferases limits potent inhibitors discovery by non-covalent binding interactions. Chi-Huey Wong and co-workers searched for *α*-1,3-fucosyltransferase (Fuc-T) inhibitors [48]. They designed a GDP-alkyne core to react with 85 azide candidates, then the GDP-triazole compounds could be directly screened for Fuc-T inhibitors in microtiter plates. Some other studies found that using an alkynyl probe and rhodamine-azide conjugate has a lower background than using a rhodamine-alkyne conjugate to label azide-tagged probes [49]. Design and synthesis of activity probes for glycosidases may be a novel approach for glycosylation studies [50]. When combined with tandem mass spectrometry, it can identify numbers of active enzymes or enzyme inhibitors.

#### 2.2.2. Glycan Microarrays

For over a decade, microarrays, a multiplexed lab-on-a-chip, have been extensively developed as a high-throughput screening method that assays large amounts of biomolecular interactions. Carbohydrate microarrays, known as “a new set of technologies at the frontier of glycomics” provides an ideal tool for glycan research [51]. The biggest challenge could be the strategies for immobilize carbohydrates on a solid surface which are strongly dependent on the linker length and the immobilization concentration [52]. Among all those developed technologies, Wong and co-workers explored the use of Cu(I)-catalyzed azide-alkyne cyclooaddition to attach oligosaccharides on micro-titer plates [53]. First, an alkynylated C_14_ hydrocarbon chain is adsorbed on the slide by hydrophobic interactions; then azido sugars are combined through CuAAC. Another way is to produce Staudinger ligation between azide tagged glycans and phosphine-derivatized plates [54].

**Figure 6:**
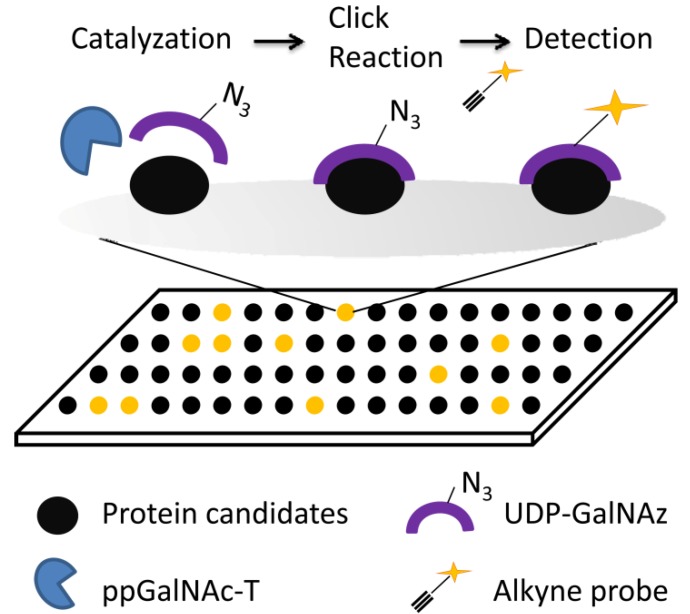
Scheme of ppGalNAc-T substrates profiling by click chemistry. Glycan enzymes catalyzed the connection of azide labeled donors to substrates. Then protein complex reacted with alkyne labeled fluorescence probe by click reaction. Substrate proteins can be detected ultimately by fluorescence scanning.

Our group established a method based on a click chemistry strategy for profiling polypeptide *N-*acetylgalactosaminyltransfemse (ppGalNAc-T) substrates on microarrays. To identify protein substrates for human ppGalNAc-Ts, we took advantage of the high-throughput capability of protein microarrays and the specificity of azido labeling, and a protein microarray-based click chemistry strategy was developed. This strategy was based on two key components, *i.e.*, a proteome microarray with ~16,000 affinity purified human proteins served as target protein reservoir, azide groups on GalNAz and the subsequent conjugation of fluorescent labeling through click chemistry served as signal readout (Figure 6).

The azide here provides us a way to immobilize carbohydrates on plates either by azide-alkyne click chemistry or Staudinger ligation, and enables us to analyze carbohydrate-protein interactions, and to indentify novel glycan-biding substrates, to promote more in-depth glycomics research. Although carbohydrate microarrays still have some limitations, such as the weakness in profiling weakly binding proteins, they have made great contributions to the development of glycomics.

## 3. Conclusion and Future Directions

As illustrated above, the exquisite selectivity and biocompatibility of bioorthogonal click chemistry render it a valuable chemical approach for biological research. Especially in glycobiology, it has brought about a fundamental revolution in glycan imaging and metabolic engineering. Among all the explored bioorthogonal reagents, azide was the most discussed and applied as a valuable handle in glycan-related studies. A major breakthrough was in the non-invasive imaging of glycans in cells, and then this was extended to live organisms, such as mice and zebrafishes.

On the basis of click chemistry study, chemists are exploiting new methods to maximize its potential, such as developing new reagents to reduce the toxicity of copper, new catalytic systems to achieve best reaction kinetics, new strategies to improve stability of azide-Staudinger ligation and so on. On the other hand, new bioorthogonal couples are under investigation as well as the improvement of existing reagents.

For azide-based bioorthogonal click chemistry in glycobiology, four directions can further be developed: (1) *The attractive features of azide as a chemical handle.* This enables immobilization of targets on solid phase media for biological manipulation, site directed reactions as well as high-throughput screening. (2) *Indentifying new bioorthogonal functional groups*. Multiple unnatural sugars enable labeling different portion of glycoms once at a time, thus provide a flexible tool to trace glycan pattern changes and pathological processes. (3) *Large-scale glycomic research*. Combined with mass spectrometry technology, azide-tagged substrates can be further purified for glycomic analysis using the advantages of metabolic engineering. This facilitates screening of disease-related glycans, glycoproteins and glycan binding proteins, discovering the discrepancy of glycoconjugates expression in disease development, and identifying biomarkers for the early diagnosis of tumor. (4) *Receptor related disease developing mechanism research*. Glycans serves as recognition molecules, which affect the receptor-binding preference of cancer cells or virus in developing diseases. Non-invasive labeling of glycans with azide enables its detection by alkyne probes or by phosphine tags, thus further facilitating the study of diseases. Click chemistry, in particular the azide-based bioorthogonal click chemistry, has demonstrated its advantages within the last 10 years. Undoubtedly, it will continue to play an irreplaceable role in glycobiology research in the future.
